# Graph contrastive learning of subcellular-resolution spatial transcriptomics improves cell type annotation and reveals critical molecular pathways

**DOI:** 10.1093/bib/bbaf020

**Published:** 2025-01-30

**Authors:** Qiaolin Lu, Jiayuan Ding, Lingxiao Li, Yi Chang

**Affiliations:** School of Artificial Intelligence, Jilin University, Qianjin Street 2699, 130010 Changchun, China; Department of Computer Science and Engineering, Michigan State University, 220 Trowbridge Rd, East Lansing, MI 48824, United States; Department, Boston University, Commonwealth Ave, Boston, MA 02215, United States; School of Artificial Intelligence, Jilin University, Qianjin Street 2699, 130010 Changchun, China; International Center of Future Science, Jilin University, Qianjin Street 2699, 130010 Changchun, China; Engineering Research Center of Knowledge-Driven Human-Machine Intelligence, Jilin University, Qianjin Street 2699, 130010 Changchun, China

**Keywords:** spatial transcriptomics, graph contrastive learning, cell type annotation, subcellular gene neighborhood network

## Abstract

Imaging-based spatial transcriptomics (iST), such as MERFISH, CosMx SMI, and Xenium, quantify gene expression level across cells in space, but more importantly, they directly reveal the subcellular distribution of RNA transcripts at the single-molecule resolution. The subcellular localization of RNA molecules plays a crucial role in the compartmentalization-dependent regulation of genes within individual cells. Understanding the intracellular spatial distribution of RNA for a particular cell type thus not only improves the characterization of cell identity but also is of paramount importance in elucidating unique subcellular regulatory mechanisms specific to the cell type. However, current cell type annotation approaches of iST primarily utilize gene expression information while neglecting the spatial distribution of RNAs within cells. In this work, we introduce a semi-supervised graph contrastive learning method called Focus, the first method, to the best of our knowledge, that explicitly models RNA’s subcellular distribution and community to improve cell type annotation. Focus demonstrates significant improvements over state-of-the-art algorithms across a range of spatial transcriptomics platforms, achieving improvements up to **27.8%** in terms of accuracy and **51.9%** in terms of F1-score for cell type annotation. Furthermore, Focus enjoys the advantages of intricate cell type-specific subcellular spatial gene patterns and providing interpretable subcellular gene analysis, such as defining the gene importance score. Importantly, with the importance score, Focus identifies genes harboring strong relevance to cell type-specific pathways, indicating its potential in uncovering novel regulatory programs across numerous biological systems.

## Introduction

Recent developments in imaging-based spatial transcriptomics (iST) [1–9] empower us to measure individual RNA molecules at subcellular resolution, which, in turn, facilitates the revelation of intricate spatial distribution of distinct cell types, and more importantly the cell type-specific subcellular localization of the transcripts. Consequently, these technologies hold exciting potential to shed light on the gene expression dynamics and regulation across many cell types at unprecedented spatial resolution. However, analyzing such data to achieve these goals will introduce several unfathomable computational hurdles. Among them, cell type annotation stands out as a particularly formidable challenge due to its low number of quantified genes, which results from the intrinsic limitation of the diffraction limit of light, the spectral overlap of the fluorescence probes, etc. For example, the Nanostring CosMx [3] platform defines the subcellular expression of merely 960 genes. Similarly, Xenium from 10$\times $ Genomics [8] measures only hundreds of genes in total. While MERFISH [7] in principle can measure the entire transcripts through a sophisticated multiplexing scheme, it usually can only robustly measure a few hundred genes. Identifying and categorizing diverse cell types within a mixed population is a crucial objective in single-cell analysis. This task is particularly challenging in spatial transcriptomics (iST) due to the low number of measured genes or high dropout rates. Deep learning techniques [10–18] have been increasingly applied to tackle these challenges. Many cell type annotation methods are designed for single-cell RNA-seq data, such as CellTypist [11], which uses logistic regression for immune cell identification, and TOSICA [12], a deep learning-based multi-head self-attention network, are designed for single-cell RNA-seq data. ACTTINN [13] employs neural networks using reference datasets to predict cell type for each cell. Furthermore, scDOT [19] leverages distance metric learning and Optimal Transport to refine cell type assignments by aligning cells across datasets while preserving biological relationships. These methods rely solely on gene expression values, which has led to the development of new approaches that also incorporate spatial information of cells. Graph-based methods like scDeepSort [14] and sigGCN [15] model complex relationships between cells using graph neural networks (GNN) [20]. sigGCN constructs gene interaction networks based on weighted adjacency matrices, and scDeepSort uses a bipartite graph to represent both cells and genes. Spatial transcriptomics-specific methods have emerged, such as spatial-ID [21], a GCN-based approach that incorporates the spatial information of cells but not of RNA transcripts, and TACCO [22], which transfers annotations from single-cell data to spatial data using optimal transport.

**Figure 1:**
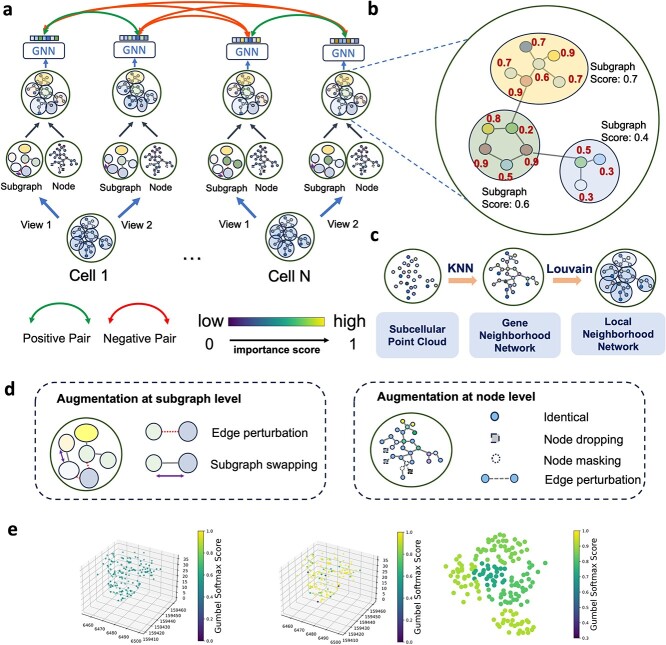
An overview of the Focus framework. (a) The graph contrastive learning workflow in Focus. (b) Learned scores for each node and subgraph after graph contrastive learning. (c) Gene neighborhood network construction from subcellular information. (d) Augmentation methods at subgraph and node level. (e) Left: subcellular point cloud of the same sample cell before training. Median: subcellular point cloud of the same sample cell after training. Right: subcellular Louvain clustering distribution of one example cell after training.

However, those methods often overlook the intracellular spatial distribution of RNA transcripts at the single molecule level, which potentially plays an important role in elucidating unique cell type-specific subcellular regulatory mechanisms. To incorporate spatial information of transcripts and their subcellular communities into cell type annotation, we propose Focus, a semi-supervised Graph Contrastive Learning (GCL)-based algorithm of iST at subcellular resolution (Fig. 1). To model the subcellular distribution and functional interaction between RNAs, Focus constructs gene neighborhood networks that constitute the basis for all downstream modeling. To learn subcellular features for each cell, we next employ GCL framework on the gene neighborhood network [23, 24] by maximizing the similarity between positive pairs from the same cell and minimizing the similarity between negative pairs from distinct cells within a common batch where the graph pairs are generated via augmented graphs through adding or removing graph nodes or edges. After the GCL process, Focus computes an importance score for every transcript and RNA subcellular community (the subnetwork formed by a subset of RNA transcripts), reflecting their importance for that cell. We find the genes with high scores tend to have significant importance and relevance to specific cell types based on detailed gene set enrichment analyses. We further demonstrate the effectiveness and robustness of Focus across various platforms in the context of cell type annotation.

Focus shows significant improvements over state-of-the-art algorithms. It outperforms the baseline methods across all test datasets, achieving up to **27.8%** higher accuracy and **51.9%** higher F1-scores. Notably, FOCUS maintains robustness, performing well even with limited labeled data, while baseline methods show a rapid performance decline. Additionally, gene ranking based on importance scores reveals that high-scoring genes are highly enriched in cell type-specific pathways. Our main contributions to this paper are as follows:

- We introduce Focus, a GCL model to enhance cell type annotation on spatial transcriptomics data by leveraging subcellular spatially resolved gene neighborhood networks. To the best of our knowledge, this is the first work that uses the spatial information of the transcript and the RNA subcellular communities in the cell to improve cell type annotation.
- Focus exhibits high robustness even when provided with a limited amount of labeled data, and outperforms state-of-the-art methods by a large margin across various iST platforms.
- Based on learned importance scores for genes by Focus, we identified novel genes with higher scores that are highly relevant to cell type-specific pathways.

## Methods

The key idea of Focus is to involve subcellular localization of RNA molecules or RNA subcellular communities within cells to learn cell type-specific intracellular spatial distribution of RNA for cell type identification under the setting of limited or even no labeled data. We achieve the goal with GCL. An overview of Focus is shown in Fig. 1. The input of Focus is gene neighborhood networks constructed from transcripts within cells. Each cell will produce two augmentation views, with each of both being generated by the combination of subgraph and node-level augmentations. Given augmented graph views of each cell, a GNN model is employed to update node embedding and to learn subgraph embedding. Focus aims to maximize the similarity between positive pairs from two augmented views of the same cell and minimize the similarity between negative pairs from different cells within a common batch guided by limited labeled data.

### Gene neighborhood network construction

To ensure the quality of the input data, we process the original subcellular spatial transcriptomics data with the following steps:

- Elimination of low-quality cells: we exclude cells that are either non-viable or exhibit a transcript count below a predefined threshold denoted as $\alpha $. The determination of the threshold $\alpha $ is dataset-specific; for the MERFISH, CosMx SMI, and Xenium datasets, values of 100, 50, and 50, respectively, are employed.
- Exclusion of negative control targets: given that negative control probes are designed to capture extraneous sequences not present in the tissue, serving as non-target controls to assess non-specific ISH probe hybridization, we filter out negative control targets and retain transcripts that corresponded to genes.

As shown in Fig. 1(c), the connections between transcripts are established based on their spatial coordinates within each cell using the K-nearest neighbors algorithm [25]. Specifically, two transcripts are connected if their separation distance is within a specified radius, denoted as $d$.

The choice of $d$ values for each sequencing technology is guided by the goal of ensuring each transcript is connected to an average of 10–30 neighbors, a range that balances graph sparsity and computational efficiency. This range is selected based on guidelines from SpaGNN [26], which demonstrates that overly sparse graphs (with small $d$) fail to capture sufficient neighborhood information, while overly dense graphs (with large $d$) increase computational complexity and may obscure spatial patterns. By maintaining this balance, we optimize graph density to support robust spatial transcriptomics analysis. For CosMx SMI, MERFISH, and Xenium, the $d$ values are chosen as 12 pixels, 2 micrometers ($\mu $m), and 3 micrometers ($\mu $m), respectively. These values reflect the resolution and spatial scales inherent to each technology. Specifically, the units of coordinate in CosMx SMI is based on pixel units, where each pixel corresponds to 0.18 $\mu $m, while MERFISH and Xenium report data directly in micrometers ($\mu $m). When converted to a consistent metric, the $d$ values become approximately 2.16, 2, and 3 $\mu $m, respectively, making the $d$ values comparable across the different sequencing technologies.

As a result, in the constructed gene neighborhood network, individual nodes represent single transcripts and are linked to an average of 10-30 neighbors. To better simulate transcript enrichment, a clustering method like Louvain [27] is employed to group the constructed graph into several local gene neighborhood networks, which refer to local RNA density or subcellular spatial regions to reveal the subcellular organization of RNA.

### Data augmentation

Previous studies [28–31] have revealed a strong correlation between the selection of specific components in a graph (such as nodes, subgraphs, etc.) and the intrinsic properties of the graph during contrastive learning. Consequently, the graph property can be effectively captured by retaining highly relevant nodes or subgraphs while eliminating less critical components [32]. Thus it’s essentially the learning process of the intracellular spatial distribution of RNA transcripts for each cell type. Based on the constructed gene neighborhood network with subgraphs, we carefully select several augmentation techniques in two levels, node level and subgraph level, as shown in Fig. 1(d):

- Augmentation at the node level: **identical** implies the preservation of edges or nodes in their original state, **node dropping** involves the removal of nodes, resulting in an alteration of the graph structure, **node masking** solely conceals node attributes while maintaining the graph structure, and **edge perturbation** pertains to the deletion of edges connecting nodes. Here, we list several data augmentation methods at the node level. **identical**, **node dropping**, **node masking** are directly related to the procession on each transcript while edge perturbation can indirectly affect each transcript by adding noise to the links between gene symbols.
- Augmentation at the subgraph level: **edge perturbation** involves the potential removal of edges between subgraphs, while **subgraph swapping** entails the exchange of two subgraphs. Since a subgraph represents a larger system than a single node, we do not use direct augmentation techniques like subgraph removal or subgraph masking that could disrupt the entire cell graph. Instead, we employ edge perturbation and subgraph swapping to indirectly affect the links within each subgraph.
- Combining augmentations at these two levels enables us to comprehensively address both local and global aspects of the data. Node-level augmentations enhance the model’s sensitivity to fine-grained transcript interactions, while subgraph-level augmentations improve its understanding of larger spatial patterns and functional groupings. This complementary approach ensures that the model can robustly analyze spatial transcriptomics data across multiple scales. Similar strategies of multi-scale graph augmentations have been shown to improve performance in various graph-based tasks, such as molecular property prediction [33, 34], drug–drug interaction networks [35], and spatial biology data analysis [36]. These studies consistently highlight the importance of addressing hierarchical structures in graph data to achieve better generalization and accuracy. Through extensive experiments (as detailed in ablation studies), we observed that combining these augmentation strategies significantly improves the model’s accuracy compared to using node-level or subgraph-level augmentations alone.

### The proposed architecture

After graph construction introduced in previous section, we have a gene neighborhood graph $G = (V, E)$, where $ V\text{=}\{v_{1},v_{2},...,v_{n}\} $ denotes a node set with $n$ transcripts in the cell, and $E$ denotes an edge set. Node feature is represented by $X\in \mathbb{R}^{n\times d}$ where the node embedding is initialized with one hot embedding with dimension $d$, and $d$ corresponds to the total count of gene symbols. The graph structure is characterized as an adjacent matrix $A \in R^{n \times n}$. The graph would be further partitioned into $k$ subgraphs $\{\mathbf{S_{1}},\mathbf{S_{2}},...,\mathbf{S_{k}}\}$ by a graph clustering algorithm like Louvain. Focus consists of multiple GIN layers to update node embeddings and subgraph embeddings. Specifically, each GIN layer is defined as follows:


(1) \begin{align*}& H_{v}^{(l+1)} = \text{ReLU} \left( \left( (1 + \epsilon^{(l)}) H_{v}^{(l)} + \sum_{u \in N(v)} H_{u}^{(l)} \right) W^{(l)} \right)\end{align*}

where $ H_{v}^{(l)} $ is the node representation of node $ v $ at the $ l $-th layer, $ \epsilon ^{(l)} $ is a learnable parameter for the $ l $-th layer, $ W^{(l)} $ is the weight matrix of the $ l $-th layer, and $N(v)$ denotes the set of neighbors of node $ v $. $\text{ReLU}(\cdot )$ is the nonlinear activation function. Here, the input for the first layer would be the original node representation $ H^{(0)} = X $.

Here, we utilize the Gumbel-Softmax strategy [37], a sampling method tailored for drawing samples from a discrete distribution in a differentiable way. This technique refines the decision-making process by determining which nodes to augment for each method during the training process. We calculate the Gumbel-Softmax probability for each node, assigning a score from 0 to 1. These scores subsequently inform the choice and implementation of augmentation techniques, guaranteeing a more precise and customized approach to data augmentation. Moreover, these scores reflect the importance of each node within the overall cellular graph:

- **Node Level**: the score of node is obtained by calculating the probability of node level augmentation, including node dropping and node masking. Specifically, given the node embedding $H_{v}$ from GIN layers, the score of node when using node dropping or masking strategy can be formulated as follows:
  (2) \begin{align*} & p_{v_{i}} = 1- \text{GumbelSoftmax}(\text{MLP}_{i}(H_{v})) \end{align*}
   
  (3) \begin{align*} & p_{v} = \sum_{i} p_{v_{i}}\end{align*}
  where $\text{MLP}_{i}$ refers to a multilayer perceptron [38] for one direct augmentation $i$ (e.g. node masking or node dropping), $H_{v}$ denotes the learned embedding of node $v$ from GIN, $p_{v}$ represents the score drawn from the distribution used to determine the nodes chosen for augmentation. For edge perturbation, the probability of each edge being maintained during the augmentation process is determined by taking the average of the scores associated with the nodes related to that edge.
- **Subgraph Level**: Here, we reweight the node-level scores within each subgraph to represent the subgraph-level score. Thus, given a subgraph **S** which contains $m$ nodes, the score of the subgraph can be formulated as follows:
  (4) \begin{align*}& p_{\mathbf{S}}=\frac{1}{m}\sum_{i=1}^{m}{p_{\mathbf{v}}}\end{align*}
  Similar to the augmentation method at the node level, for edges between subgraphs, we determine the probability of these edges being retained during the augmentation process by calculating and weighting the scores of all related nodes associated with these edges. Specifically, in the process of subgraph swapping augmentation, for subgraphs that have already been augmented, we randomly sample nodes from the subgraphs that are set to be connected next in order to establish new inter-subgraph connections.

Upon completing the augmentation process at both the node level and subgraph level, we introduce the augmented graph into ResGCN [39] for the ongoing update of node embeddings and edge features. Ultimately, the graph embedding is obtained as $H_{G}=\sum _{v=1}^{n^{^{\prime}}}H_{v}^{(t)}$, where $n^{^{\prime}}$ represents the number of nodes in the augmented graph, $H_{v}^{(t)}$ is the node embedding at the $t$-th layer of ResGCN.

### Training process

In order to better learn the property of each cell, we follow the InfoMin principle [40] which states that good contrastive learning should maximize the label-related information while reducing the similarity between them. In the training phase, a data batch comprising N cells is randomly selected, and this batch is fed into the two graph view generators to generate 2N graph views. The two augmented views originating from the same input graph are treated as the positive view pair, while the views derived from distinct input graphs are considered the negative pair. In our work, we define contrastive learning loss $L_{cl}$ to maximize the uniformity between positive pairs and negative pairs. To be specific, we adopt the normalized temperature-scaled cross-entropy loss(NT-XEnt) $L_{cl}$, which can be formulated as follows:


(5) \begin{align*} & \ell_{i,j}=-\log\frac{\exp(\text{sim}(\mathbf{H}_{Gi},\mathbf{H}_{Gj})/\tau)}{\sum_{k=1}^{2N}\mathbb{I}\{k\neq i\}\text{exp}(\text{sim}(\mathbf{H}_{Gi},\mathbf{H}_{Gj})/\tau)} \end{align*}

(6) \begin{align*} & L_{cl}=\frac{1}{2N}\sum_{k=1}^{N}[\ell(2k-1,2k)+\ell(2k,2k-1)]\end{align*}

Here, ($i$, $j$) represents a positive pair of samples from the same cell, while ($i$, $k$) denotes a randomly sampled pair from the batch. $\mathbf{H}_{Gi}$ denotes the embedding of cell $i$ obtained from the final output of ResGCN. The cosine similarity between cell $i$ and cell $k$, denoted as $\text{sim}{(\mathbf{H}_{Gi},\mathbf{H}_{Gj})} = \frac{\boldsymbol{H}_{Gi}\cdot \boldsymbol{H}_{Gj}}{\left \|\boldsymbol{H}_{Gi}\right \|_{2}\cdot \left \|\boldsymbol{H}_{Gj}\right \|_{2}}$, where $\tau $ represents the temperature parameter. The loss is computed collectively across all positive pairs within a batch. Different from other supervised learning methods that only train on reference labeled data and infer on query data,

We design a novel semi-supervised learning setting that effectively learns intrinsic properties of the query data using labeled data. Specifically, we employ a dual approach: unsupervised contrastive learning to organize the latent space based on similarities in the query and reference datasets, and supervised learning with the labeled reference dataset to ensure the model associates latent features with specific cell types. Positive and negative pairs are constructed from the query dataset to capture intrinsic relationships, while the labeled reference dataset provides biologically meaningful supervision, enabling the model to learn both general representations and cell type-specific features.

Here, we adopt the cross entropy loss $L_{cls}$ as supervised classification loss, which can be formulated as follows:


(7) \begin{align*}& L_{cls} =-\sum_{k=1}^{N}y\text{log}(\hat{y})\end{align*}

where $y$ and $\hat{y}$ denote ground truth labels and predicted labels, respectively. The overall objective $L$ of Focus can be formulated as follows:


(8) \begin{align*}& L = L_{cl} + \lambda L_{cls}\end{align*}

where $\lambda $ is a hyperparameter to balance unsupervised contrastive learning loss $L_{c l}$ and supervised classification loss $L_{c l s}$.

### Hyperparameter selection

We optimized hyperparameters using a systematic grid search approach. For the unsupervised contrastive learning module, the temperature parameter ($\tau $) was tuned in [0.1, 0.2, 0.5, 1.0], batch size in [16, 32, 64], and learning rate in [1 $\times $  $10^{-3}$, 5 $\times $  $10^{-4}$, 1 $\times $  $10^{-4}$]. For the cell type classification module, dropout rate was selected from [0.1, 0.3, 0.5], $\lambda $ in [0.5, 1, 5], and hidden layer size was tested at [16, 32, 64]. The optimal configuration, minimizing both contrastive loss and cell type classification loss, was $\tau $ = 0.5, batch size = 32, learning rate = 5 $\times 10^{-4}$, dropout rate = 0.3, $\lambda $=1 or 5, and hidden layer size = 128. Hyperparameter tuning revealed that $\tau $ = 0.5 provided the best balance between feature separation and convergence stability, while the batch size of 32 and learning rate of 5 $\times 10^{-4}$ ensured diversity and stable gradient updates. For the cell type classification module, the dropout rate (0.3) effectively prevented overfitting, and the hidden layer size (128) captured complex relationships without adding excessive complexity. Additionally, $\lambda $ values of 1 and 5 produced comparable results, indicating robustness to variations in this parameter. This systematic tuning process ensured optimal performance, generalization across datasets, and enhanced reproducibility.

## Results

### Datasets and evalutaion metrics

**Datasets.** CosMx SMI, MERFISH, and Xenium are advanced spatial transcriptomics technologies that map gene expression within tissues. CosMx SMI uses cyclic single-molecule FISH, MERFISH employs barcoded FISH, and Xenium utilizes targeted in situ sequencing or high-plex FISH for high-resolution RNA detection. Despite differences in detection methods and capabilities, all achieve accurate cell type identification through standardized processing steps like normalization, clustering, and annotation. To demonstrate the performance of Focus on cell type annotation, we collect a variety of subcellular spatial transcriptomic datasets from NanoString CosMx SMI [3], Xenium from 10$\times $ Genomics [8] and MERFISH [7] for evaluation. The tissues under evaluation encompass the human kidney, lung, breast, and mouse cortex.

- *CosMx Human Lung and Kidney*. These datasets are generated using a 960-plex CosMx RNA panel with CosMx SMI. The lung dataset consists of eight samples from non-small cell lung cancer (NSCLC) tissues, including three female and two male patients. A total of 766 313 cells were analyzed, with an average of 265 transcripts per cell, each represented by a subset of 960 selected genes. Each cell was classified into one of 18 unique cell types. For the kidney dataset, five samples were curated from kidney core biopsy tissues of lupus nephritis patients, collected from three different patients. These samples were also processed using the CosMx SMI platform, and we assign eight unique cell types for classification.
- *MERFISH Mouse Primary Motor Cortex (MOp)*. This dataset comprises spatially resolved gene expression profiles of individual cells within the mouse primary motor cortex, obtained through MERFISH. The dataset encompasses approximately 300 000 cells and 258 gene symbols, with an average of 614 transcripts detected per cell. Furthermore, it identifies a total of 24 distinct cell types within the mouse primary motor cortex.
- *Xenium Ductal carcinoma In Situ (DCIS)*. This dataset was created using Xenium from 10$\times $ Genomics and was collected from human breast tissue containing DCIS tumor cells. We conduct data cleaning and utilized two replicates from a single sample, resulting in the detection of approximately 300 000 cells and 313 genes. The dataset encompasses a total of 19 distinct cell types.

**Evaluation metrics.** To assess the effectiveness of cell type annotation, we adopt the evaluation metrics *Accuracy* and *F1-score* following previous cell type annotation studies [11–14]. We obtain the ground truth released from original datasets and then calculate the *Accuracy* and *F1-score* between ground truth and predicted cell type labels generated by Focus or baselines. The *Accuracy* and *F1-score* are formulated as follows:


(9) \begin{align*} & \mathit{Accuracy} =\frac{\text{TP}+\text{TN}}{\text{TP}+\text{FP}+\text{TN}+\text{FN}} \end{align*}

(10) \begin{align*} & \mathit{F1-score} =\frac{2 \times \text{TP}}{2 \times \text{TP}+\text{FP}+\text{FN}}\end{align*}

where true positive (TP) refers to correctly classified positive samples, true negative (TN) to correctly classified negative samples, false positive (FP) to samples incorrectly classified as positive, and false negative (FN) to samples incorrectly classified as negative.

### Focus achieves SOTA on cell type annotation across diverse iST platforms

To validate the efficacy of our Focus across diverse iST platforms, we perform validation experiments of cell type annotation on four datasets using Focus and state-of-the-art methods. These experiments encompass three iST platforms: CosMx, MERFISH and Xenium. For each dataset, we averaged the results from three experimental runs conducted on several sample pairs, with one sample designated as the reference data and another as the query data. More details can be found in Supplementary Tables 4–8. During the training of Focus, query data without label is also included and contributes informative data for contrasting and understanding the inherent characteristics of the query data. In practical scenarios, reference and query datasets may originate from various studies or different subjects, potentially introducing batch effects that can impair cell type annotation performance. Hence, we conduct our experiments under two conditions: utilizing samples from the same subject and samples from distinct subjects for comprehensive evaluation. We also benchmark scDeepSort [14], CellTypist [11], TOSICA [12], ACTINN [13], classical cell type annotation methods on single-cell RNA seq data and Tacco [22], which is specifically designed for spatially transcriptomic data on the above datasets for comparison.

As shown in Table 1 and Table 2, we observe that regardless of whether the reference and query data are sourced from the same or distinct subjects, our Focus consistently outperforms state-of-the-art approaches both on accuracy and F1-score perspectives. Notably, on the Xenium DCIS dataset, Focus demonstrates a remarkable advantage, surpassing the second-ranked method by a substantial **27.8%** in terms of accuracy and **50%** in terms of F1-score. To be specific, Focus achieves 87.1% (accuracy) and 71.2% (F1-score) while the second-ranked method ACTINN gets 59.3% (accuracy) and 19.3% (F1-score) on Xenium DCIS dataset, The methods specialized in annotating single-cell data, such as CellTypist and scDeepSort, frequently employ the filtration of genes with exceedingly low expression levels in cells. Yet, this strategy can potentially constrain their capacity to harness the full limited gene information within spatial transcriptomics data. Tacco is specifically designed for spatial transcriptomics data, and it performs well in capturing cell-level spatial information on MERFISH and CosMx Lung datasets. However, when applied to other datasets featuring intricate cell types, it encounters notable variability in cell type prediction due to the complexity of cell type. Instead, our Focus focuses on acquiring cell type-specific information at the subcellular level, and this capability is not constrained by the quantity of cell types present. As a result, we consistently achieve top-tier performance, regardless of whether the dataset contains a limited or extensive variety of cell types.

**Table 1:** Performance of different methods on CosMx Lung, CosMx Kidney, and MERFISH MOp datasets. The reference and query samples come from the same patient or mouse.

**Dataset**	**CosMx Lung**		**CosMx Kidney**		**MERFISH MOp**		**Xenium DCIS**	
**Model**	**Accuracy**	**F1-score**	**Accuracy**	**F1-score**	**Accuracy**	**F1-score**	**Accuracy**	**F1-score**
**Focus**	0.904 $\pm $ 0.009	0.704 $\pm $ 0.027	**0.688 $\pm $ 0.013**	**0.694 $\pm $ 0.006**	**0.948 $\pm $ 0.023**	**0.909 $\pm $ 0.082**	**0.871 $\pm $ 0.006**	**0.712 $\pm $ 0.030**
**scDeepSort**	0.725 $\pm $ 0.026	0.391 $\pm $ 0.012	0.377 $\pm $ 0.005	0.248 $\pm $ 0.007	0.868 $\pm $ 0.052	0.685 $\pm $ 0.025	N/A	N/A
**CellTypist**	0.634 $\pm $ 0.018	0.429 $\pm $ 0.013	0.478 $\pm $ 0.028	0.495 $\pm $ 0.010	N/A	N/A	0.371 $\pm $ 0.011	0.144 $\pm $ 0.002
**TOSICA**	0.854 $\pm $ 0.021	0.638 $\pm $ 0.001	0.668 $\pm $ 0.002	0.664 $\pm $ 0.002	0.942 $\pm $ 0.004	0.885 $\pm $ 0.059	0.381 $\pm $ 0.044	0.176 $\pm $ 0.005
**ACTTINN**	0.852 $\pm $ 0.002	0.566 $\pm $ 0.029	0.569 $\pm $ 0.009	0.551 $\pm $ 0.023	0.938 $\pm $ 0.011	0.853 $\pm $ 0.059	0.593 $\pm $ 0.013	0.193 $\pm $ 0.004
**Tacco**	**0.916 $\pm $ 0.012**	**0.729 $\pm $ 0.004**	0.530 $\pm $ 0.030	0.522 $\pm $ 0.016	0.939 $\pm $ 0.011	0.902 $\pm $ 0.047	0.525 $\pm $ 0.001	0.207 $\pm $ 0.007
**scDot**	0.844 $\pm $ 0.009	0.637 $\pm $ 0.029	0.481 $\pm $ 0.028	0.506 $\pm $ 0.033	0.922 $\pm $ 0.029	0.868 $\pm $ 0.080	N/A	N/A

Bold text indicates the best, underlined text indicates the second-best, and ‘N/A’ means no meaningful results.

**Table 2:** Performance of different methods on CosMx Lung, CosMx Kidney, and MERFISH MOp datasets. The reference and query samples come from the different patients or mice.

**Dataset**	**CosMx Lung**		**CosMx Kidney**		**MERFISH MOp**	
**Model**	**Accuracy**	**F1-score**	**Accuracy**	**F1-score**	**Accuracy**	**F1-score**
**Focus**	**0.692 $\pm $ 0.119**	**0.399 $\pm $ 0.173**	**0.715 $\pm $ 0.025**	**0.708 $\pm $ 0.020**	**0.934 $\pm $ 0.010**	**0.865 $\pm $ 0.048**
**scDeepSort**	0.462 $\pm $ 0.059	0.150 $\pm $ 0.045	0.173 $\pm $ 0.119	0.065 $\pm $ 0.062	0.854 $\pm $ 0.033	0.680 $\pm $ 0.071
**CellTypist**	0.450 $\pm $ 0.112	0.214 $\pm $ 0.018	0.450 $\pm $ 0.066	0.472 $\pm $ 0.062	N/A	N/A
**TOSICA**	0.634 $\pm $ 0.202	0.386 $\pm $ 0.158	0.709 $\pm $ 0.002	0.703 $\pm $ 0.017	0.925 $\pm $ 0.006	0.825 $\pm $ 0.006
**ACTTINN**	0.636 $\pm $ 0.047	0.299 $\pm $ 0.057	0.584 $\pm $ 0.009	0.543 $\pm $ 0.023	0.931 $\pm $ 0.008	0.832 $\pm $ 0.039
**Tacco**	0.640 $\pm $ 0.080	0.363 $\pm $ 0.146	0.575 $\pm $ 0.049	0.561 $\pm $ 0.056	0.922 $\pm $ 0.011	0.850 $\pm $ 0.034
**scDot**	0.663 $\pm $ 0.134	0.390 $\pm $ 0.174	0.584 $\pm $ 0.062	0.554 $\pm $ 0.069	0.918 $\pm $ 0.001	0.847 $\pm $ 0.047

Bold text indicates the best, underlined text indicates the second-best, and ‘N/A’ means no meaningful results.

It is worth noting that on rather homogeneous datasets Focus still achieves superior performance (71.5% accuracy for the first setting) compared with other methods (scDeepSort: 17.3%, Tacco: 57.5%, CellTypist: 45.0%, TOSICA: 70.9%, ACTINN: 58.4%, and scDot: 58.4%) on CosMx kidney dataset. Among this dataset, there are three main cell types: proliferating proximal tubule cell, distinct proximal tubule 2 cell and distinct proximal tubule 1 cell, which are very similar to each other. This brings challenges to cell type annotation and these cell types are very difficult to distinguish by previous biological researches [41, 42], which mainly rely on gene expression values. The paramount aspect of Focus lies in the acquisition of cell type information from RNA’s subcellular spatial molecular pattern, which proves to be more effective when compared with methods relying solely on gene expression values. Furthermore, Focus presents remarkable transferability, even when applied to samples from distinct subjects. In contrast, other approaches experience a performance decline when confronted with reference data from different subjects. This heightened challenge in spatial transcriptomics data, characterized by a shallower read depth, presents difficulties in feature learning at the individual cell level. However, our model consistently attains the highest classification performance across all datasets.

### Ablation studies

As shown in Table 3, we conduct ablation studies on CosMx Lung, Xenium DCIS, MERFISH MOp datasets to evaluate the impact of different data augmentation strategies on the performance of Focus. The study assessed augmentation techniques at both the node and subgraph levels, as well as their combinations. Node-level augmentation, such as node dropping, feature masking, or altering connections, significantly improved model robustness and generalization by introducing variability. Subgraph-level augmentation, which perturbs entire subgraphs, showed marginal gains compared to node-level augmentation. The combination of both node- and subgraph-level augmentations proved most effective, yielding the highest accuracy and F1-scores across nearly all the platforms.

**Table 3:** Ablation studies on CosMx Lung, Xenium DCIS, MERFISH MOp datasets.

**Augmentation**	**Augmentation**	**CosMx Lung**		**Xenium DCIS**		**MERFISH MOp**	
**Level**	Type^*^	**Accuracy**	**F1-score**	**Accuracy**	**F1-score**	**Accuracy**	**F1-score**
**Node**	**Identical**	0.852 $\pm $ 0.044	0.663 $\pm $ 0.023	0.838 $\pm $ 0.012	0.701 $\pm $ 0.021	0.947 $\pm $ 0.032	0.911 $\pm $ 0.021
**Node**	**ND**	0.872 $\pm $ 0.021	0.710 $\pm $ 0.017	0.842 $\pm $ 0.019	0.729 $\pm $ 0.021	0.970 $\pm $ 0.015	0.961 $\pm $ 0.018
**Node**	**NM**	0.863 $\pm $ 0.018	0.698 $\pm $ 0.018	0.832 $\pm $ 0.020	0.711 $\pm $ 0.019	0.954 $\pm $ 0.009	0.922 $\pm $ 0.010
**Node**	**EP**	0.869 $\pm $ 0.011	0.686 $\pm $ 0.014	0.834 $\pm $ 0.007	0.707 $\pm $ 0.006	0.967 $\pm $ 0.021	0.944 $\pm $ 0.011
**Node**	**ND & NM**	0.882 $\pm $ 0.015	0.699 $\pm $ 0.012	0.855 $\pm $ 0.011	0.731 $\pm $ 0.023	0.944 $\pm $ 0.014	0.887 $\pm $ 0.025
**Node**	**ND, NM & EP**	**0.901 $\pm $ 0.013**	0.691 $\pm $ 0.021	**0.871 $\pm $ 0.018**	0.733 $\pm $ 0.024	0.965 $\pm $ 0.017	0.921 $\pm $ 0.023
**Subgraph**	**EP**	0.857 $\pm $ 0.029	0.671 $\pm $ 0.030	0.852 $\pm $ 0.032	0.711 $\pm $ 0.021	0.937 $\pm $ 0.033	0.889 $\pm $ 0.029
**Subgraph**	**SW**	0.873 $\pm $ 0.016	0.681 $\pm $ 0.031	0.858 $\pm $ 0.028	0.732 $\pm $ 0.022	0.945 $\pm $ 0.018	0.889 $\pm $ 0.019
**Subgraph**	**EP & SW**	0.862 $\pm $ 0.022	0.672 $\pm $ 0.028	0.856 $\pm $ 0.026	0.700 $\pm $ 0.018	0.941 $\pm $ 0.026	0.885 $\pm $ 0.024
**Node & Subgraph**	**ALL**	**0.901 $\pm $ 0.021**	**0.712 $\pm $ 0.008**	0.867 $\pm $ 0.019	**0.734 $\pm $ 0.021**	**0.975 $\pm $ 0.011**	**0.967 $\pm $ 0.018**

^*^Identical: no augmentation; ND: node dropping; NM: node masking; EP: edge perturbation; SW: subgraph swapping; ALL: all augmentations including ND, NM & EP from node level and EP & SW from subgraph level.

Bold text indicates the best and underlined text indicates the second-best.

### Graph-based gene importance scores reveals enriched cell type-specific pathways

**Figure 2:**
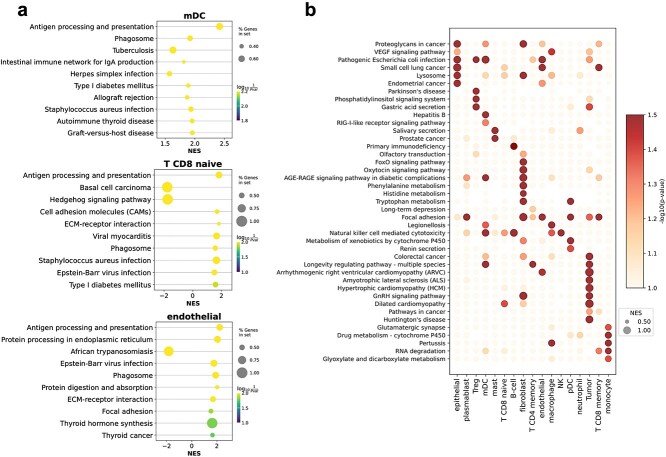
Enriched cell type-specific pathways based on gene important score from GSEA Preranked analysis. (a) Enriched pathway of mDC, CD8+ naive T cell, and endothelial cell. (b) Heatmap of selected cell type-specific pathway. NES: normalized enrichment score.

By leveraging the Gumbel-Softmax strategy for selective augmentations on each gene or transcript, we can derive scores for all genes, serving as indicators of their importance within data augmentation. To effectively illustrate the importance of each gene with respect to each cell type, we compile scores for all 960 genes, categorizing them by cell type within a dataset of 20 645 sampled cells from CosMx Lung NSCLC. It is noteworthy to emphasize that these genes are not only associated with cell types but also play roles in various biological processes, including those related to antigen processing and presentation, ECM-receptor interaction, among others. Moreover, we carefully rank these genes according to their score distribution (based on the averaged score). Based on the ranking and scores of these genes, we perform GSEA preranked gene set enrichment analysis using the gseapy package [43] and KEGG database [44]. We filter for pathways that are significantly enriched in high-ranking genes judged by setting Normalized p-val (NOM p-value) < 0.05, False Positive Rate q-value (FDR) < 0.25, and Normalized Enrichment Score (NES) $\geq $ 1, as reported by previous work [45, 46]. We find that those genes in the enriched pathways are mostly the ones with higher scores, and those pathways are highly relevant to the corresponding cell types. Given the fact that the CosMx dataset contains tumor cells and a large number of immune cells, as shown in Fig. 2(a), our enrichment analysis reveals that these cells predominantly exhibit functions highly related to antigen processing and presentation, ECM-receptor interaction, Epstein-Barr virus infection, which is consistent with their fundamental immunity functionalities. To better discern functional differences among different cell types, we select pathway information that is highly significant only in specific cell types as shown in Fig. 2(b). For NK cells, we have successfully observed highly significant signal pathways expressed within NK cells, such as natural killer cell mediated cytotoxicity. Similarly, we have identified signal pathways associated with tumor cells triggered by colorectal cancer, hypertrophic cardiomyopathy (HCM), and amyotrophic lateral sclerosis, which are consistent with previous research [47, 48]. For B cells, we have discovered specific immunological pathways, such as primary immunodeficiency, an immune deficiency disorder primarily associated with B cell dysfunction [49]. These examples convincingly demonstrate that Focus can learn pathway information highly relevant to cell types, which plays a crucial role in cell type classification for spatial transcriptomics data.

### Subgraph sampling to illustrate gene neighborhood relation

**Figure 3:**
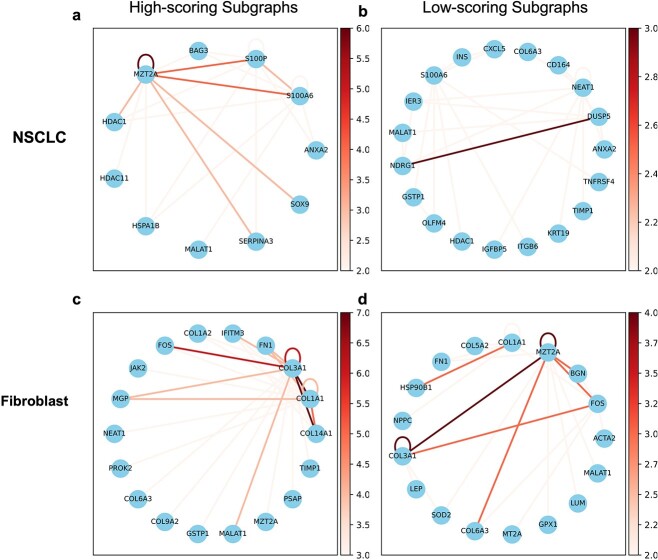
Gene-neighbor relationship diagram in different subgraphs in CosMx lung dataset. (a) Gene neighborhood network in high-scoring subgraphs of NSCLC cells. (b) Gene neighborhood network in low-scoring subgraphs of NSCLC cells. (c) Gene Neighborhood Network in high-scoring subgraphs of Fibroblast cells. (d) Gene Neighborhood Network in low-scoring subgraphs of Fibroblast cells.

Here we adopt weighted probability in Equation 4, to determine the score of the subgraph. We use the CosMx lung dataset which is also used in previous section. Based on the scores of the subgraphs, we identified the highest and lowest-scoring subgraphs for each cell type. The subgraphs with the highest scores tend to retain their structural characteristics during the contrastive learning augmentation process. Initially, we analyzed the distribution of various genes across different subgraphs. However, the distribution of the top-ranked genes in different subgraphs did not show significant differences. Additionally, previous studies have demonstrated that even within the same cell, the distribution of neighboring genes can vary across different regions. Despite the gene neighborhood network being influenced by various factors such as cell type, intracellular location, and cell state, our model indicates that higher-scoring subgraphs exhibit a more significant coexpression phenomenon and interaction network specific to certain cell types. For each cell type, we collected the highest and lowest-scoring subgraphs and analyzed the neighboring genes around each transcript. To minimize statistical errors caused by data noise, we set a threshold value of alpha = 1, removing edges where the co-occurrence frequency of genes was less than alpha. To better illustrate the subgraph scores related to each cell type, we list several high-scoring and low-scoring subgraphs associated with different cell types. In our study, we collected 1869 NSCLC cells and 1222 fibroblast cells and drew gene neighborhood network for each cell type in Fig. 3. In NSCLC cells, high-scoring subgraphs exhibit stronger neighbor connections with MZT2A, S100A6, and S100P compared to low-scoring subgraphs. MZT2A is a key gene involved in mitotic spindle formation and promotes cell proliferation [50], while S100A6 and S100P are well-known marker genes for tumor cells [51, 52]. While in the low-scoring subgraphs, DUSP5 and NDRG1 show strong connections. Many studies [53–55] have shown that DUSP5 and NDRG1 are tumor suppressors that can effectively inhibit the proliferation and metastasis of tumor cells. This aligns with the characteristic of extensive proliferation in tumor cells. In fibroblast cells, compared to low-scoring subgraphs, we found that high-scoring subgraphs exhibit strong neighbor connections among Collagen family genes such as COL9A2, COL3A1, COL1A2, COL1A1, COL14A1, and COL6A3. These Collagen genes can produce collagen proteins [56], which are crucial components of the extracellular matrix (ECM). This is consistent with the primary function of fibroblast cells. The analysis of high-scoring and low-scoring subgraphs provides a unique lens to investigate gene neighborhood relationships in spatial transcriptomics. High-scoring subgraphs often capture key gene interactions that drive critical cellular processes, such as proliferation and extracellular matrix remodeling, while low-scoring subgraphs highlight less active or potentially suppressed pathways. By comparing these subgraphs across different cell types, we can uncover functional gene clusters specific to cell states or microenvironments. This dual perspective not only enhances our understanding of cellular heterogeneity but also offers a framework for identifying potential biomarkers and therapeutic targets.

### Focus is robust to limited reference data

**Figure 4:**
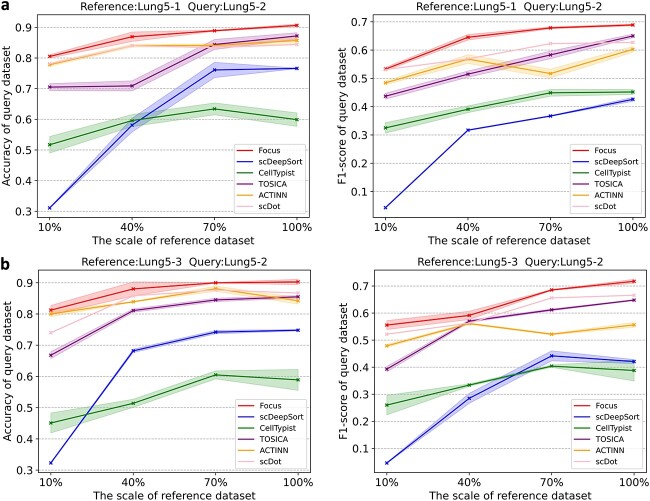
Robustness of Focus and other methods to the reference data size. The horizontal axis represents the proportion of the query dataset size relative to the reference dataset size. The reference dataset contains a total of 10 000 cells, and the query dataset size is scaled accordingly (e.g., 10%, 40%, 70%, and 100% of the reference dataset size). (a) Reference data: CosMx Lung5-1, Query data: CosMx Lung5-2. (b) Reference data: CosMx Lung5-3, Query data: CosMx Lung5-2.

Recognizing the challenge of acquiring extensive high-quality labeled reference data, we carry out experiments with a restricted pool of labeled reference data to assess the performance of each method. To achieve this, we devised four distinct scenarios where the query data remained constant at 10 000 cells while varying the proportions of the reference data to 10%, 40%, 70%, and 100% of the query dataset size. In order to ensure consistent findings and mitigate the influence of random variations, we systematically conduct a series of experiments across multiple samples on CosMx Lung dataset (Fig. 4). Our consistent observation is that, regardless of whether our Focus is trained with 10%, 40%, 70%, or 100% reference data, the variation in reference data proportion has a minimal impact on query performance, and accuracy consistently remains above 80%. However, all other methods exhibit a performance decline as reference data decreases, albeit to varying degrees. For instance, scDeepSort experiences the most significant performance drop across all experiments, while cellTypist is less affected by data reduction but consistently demonstrates lower accuracy across reference data scales. ACTINN appears less affected overall. To sum up, our Focus maintains an 80% accuracy level across various experiments, demonstrating robustness to reference data downsampling from 9k to 1k, as well as to the variations in reference data, thus underscoring its robustness.

## Discussion

In this study, we present the Focus method, which significantly enhances cell type annotation by leveraging subcellular spatial gene neighborhood networks. Our method demonstrates superior performance compared to state-of-the-art approaches across various iST platforms, including CosMx, MERFISH, and Xenium. Specifically, Focus achieves up to a 10% increase in accuracy, even with limited labeled data. This underscores the robustness and versatility of our method in diverse experimental settings.

One of the key innovations of Focus is its ability to utilize transcript’s subcellular and spatial community information. This novel approach allows for a more nuanced understanding of cellular architecture and gene expression patterns, contributing to more accurate cell type annotations. However, it is important to acknowledge that subcellular data inherently contains more noise compared to single-cell data. Additionally, the subcellular spatial mechanisms of different cell types are not yet fully elucidated, which poses challenges for interpreting complex spatial gene expression patterns.

However, our study is not without limitations. It is important to acknowledge that subcellular location of image-based spatial transcriptomics of often comes with noise. Additionally, the subcellular spatial mechanisms of different cell types are not yet fully elucidated, which poses challenges for interpreting complex spatial gene expression patterns. The noise in subcellular data can sometimes obscure true biological signals, and our current understanding of subcellular spatial mechanisms is limited. Future research of Focus should focusing on refining data preprocessing techniques to mitigate noise and developing more sophisticated models to better capture subcellular spatial dynamics. Moreover, expanding the application of Focus to a broader range of cell types and tissues will be crucial for validating its generalizability and robustness.

In conclusion, Focus represents a valuable advancement in cell type annotation methodologies, offering a powerful tool for the analysis of subcellular spatial transcriptomics data. By addressing the current limitations and further exploring the potential of spatial gene neighborhood networks, we can pave the way for deeper insights into cellular organization and function. This, in turn, will enhance our understanding of tissue heterogeneity and disease pathology, ultimately contributing to advancements in biomedical research and precision medicine.

**Key Points**

- A graph contrastive learning model (Focus) is proposed to enhance cell type annotation on spatial transcriptomics data by leveraging subcellular spatially resolved gene neighborhood networks.
- The effectiveness and robustness of our model are verified by a series of experiments on several datasets.
- Our model uncovers cell-type-specific pathways by leveraging the learned importance scores for genes.

## Supplementary Material

supplementary_bbaf020

supplementary_bbaf020

## Data Availability

Focus is freely accessible at https://github.com/OmicsML/focus. CosMx SMI data are available at https://nanostring.com /products/cosmx-spatial-molecular-imager/ffpe-dataset/. MERFISH MOP data are available at https://download.brainimagelibrary.org/cf/1c/cf1c1a431ef8d021/. Xenium Human DCIS data are available at https://www.10xgenomics.com/products/xenium-in-situ/preview-dataset-human-breast.

## References

[1] Rao A, Barkley D, França GS. et al. Exploring tissue architecture using spatial transcriptomics. Nature 2021;596:211–20. 10.1038/s41586-021-03634-9. 34381231 PMC8475179

[2] Tian L, Chen F, Macosko EZ. The expanding vistas of spatial transcriptomics. Nat Biotechnol 2023;41:773–82. 10.1038/s41587-022-01448-2. 36192637 PMC10091579

[3] He S, Bhatt R, Brown C. et al. High-plex imaging of RNA and proteins at subcellular resolution in fixed tissue by spatial molecular imaging. Nat Biotechnol 2022;40:1794–806. 10.1038/s41587-022-01483-z. 36203011

[4] Zhang M, Eichhorn SW, Zingg B. et al. Spatially resolved cell atlas of the mouse primary motor cortex by merfish. Nature 2021;598:137–43. 10.1038/s41586-021-03705-x. 34616063 PMC8494645

[5] Eng C-HL, Lawson M, Zhu Q. et al. Transcriptome-scale super-resolved imaging in tissues by RNA seqFISH+. Nature 2019;568:235–9. 10.1038/s41586-019-1049-y. 30911168 PMC6544023

[6] Alon S, Goodwin DR, Sinha A. et al. Expansion sequencing: spatially precise in situ transcriptomics in intact biological systems. Science 2021;371:eaax2656. 33509999 10.1126/science.aax2656 PMC7900882

[7] Chen KH, Boettiger AN, Moffitt JR. et al. Spatially resolved, highly multiplexed RNA profiling in single cells. Science 2015;348:aaa6090. 10.1126/science.aaa6090. 25858977 PMC4662681

[8] Janesick A, Shelansky R, Gottscho AD. et al. High resolution mapping of the tumor microenvironment using integrated single-cell, spatial and in situ analysis. Nat Commun 2023;14:8353. 10.1038/s41467-023-43458-x. 38114474 PMC10730913

[9] Lan W, He G, Liu M. et al. Transformer-based single-cell language model: a survey. Big Data Min Anal 2024;7:1169–86. 10.26599/BDMA.2024.9020034.

[10] Molho D, Ding J, Tang W. et al. Deep learning in single-cell analysis. ACM Transactions on Intelligent Systems and Technology 2024;15:1–62. 10.1145/3641284.

[11] Domínguez Conde C, Xu C, Jarvis LB. et al. Cross-tissue immune cell analysis reveals tissue-specific features in humans. Science 2022;376:eabl5197. 10.1126/science.abl5197. PMC7612735 35549406

[12] Chen J, Xu H, Tao W. et al. Transformer for one stop interpretable cell type annotation. Nat Commun 2023;14:223. 10.1038/s41467-023-35923-4. PMC9840170 36641532

[13] Ma F, Pellegrini M. ACTINN: automated identification of cell types in single cell RNA sequencing. Bioinformatics 2020;36:533–8. 10.1093/bioinformatics/btz592. 31359028

[14] Shao X, Yang H, Zhuang X. et al. scDeepSort: a pre-trained cell-type annotation method for single-cell transcriptomics using deep learning with a weighted graph neural network. Nucleic Acids Res 2021;49:e122–2. 10.1093/nar/gkab775. 34500471 PMC8643674

[15] Wang T, Bai J, Nabavi S. Single-cell classification using graph convolutional networks. BMC Bioinform 2021;22:1–23. 10.1186/s12859-021-04278-2. PMC8268184 34238220

[16] Shen R, Liu L, Zihan W. et al. Spatial-ID: a cell typing method for spatially resolved transcriptomics via transfer learning and spatial embedding. Nat Commun 2022;13:7640. 10.1038/s41467-022-35288-0. 36496406 PMC9741613

[17] Lan W, Chen J, Liu M. et al. Deep imputation bi-stochastic graph regularized matrix factorization for clustering single-cell RNA-sequencing data. IEEE/ACM Trans Comput Biol Bioinform 2024; pp. 1–11. 10.1109/TCBB.2024.3387911. 38607719

[18] Lan W, Liu M, Chen J. et al. JLONMFSC: clustering scRNA-seq data based on joint learning of non-negative matrix factorization and subspace clustering. Methods 2024;222:1–9. 10.1016/j.ymeth.2023.11.019. 38128706

[19] Xiong Y-X, Zhang X-F. scDOT: enhancing single-cell RNA-seq data annotation and uncovering novel cell types through multi-reference integration. Brief Bioinform 2024;25:bbae072. 38436563 10.1093/bib/bbae072 PMC10939303

[20] Kipf TN, Welling M. Semi-supervised classification with graph convolutional networks. 5th International Conference on Learning Representations 2017. arXiv preprint arXiv:1609.02907. 2016.

[21] Hinton GE, Salakhutdinov RR. Reducing the dimensionality of data with neural networks. Science 2006;313:504–7. 10.1126/science.1127647. 16873662

[22] Mages S, Moriel N, Avraham-Davidi I. et al. TACCO unifies annotation transfer and decomposition of cell identities for single-cell and spatial omics. Nat Biotechnol 2023;41:1465–73. 10.1038/s41587-023-01657-3. PMC10513360 36797494

[23] Chen T, Kornblith S, Norouzi M. et al. A simple framework for contrastive learning of visual representations. In: International Conference on Machine Learning conference, PMLR, pp. 1597–607. 2020.

[24] Wen H, Ding J, Jin W. et al. Graph neural networks for multimodal single-cell data integration. Proceedings of the 28th ACM SIGKDD Conference on Knowledge Discovery and Data Mining, 2022, 4153–63. 10.1145/3534678.3539213.

[25] Dong W, Moses C, Li K. Efficient k-nearest neighbor graph construction for generic similarity measures. In: Proceedings of the 20th international conference on World wide web, Association for Computing Machinery, New York, NY, United States, pp. 577–86, 2011.

[26] Fang Z, Ford AJ, Thomas H. et al. Subcellular spatially resolved gene neighborhood networks in single cells. Cell Rep Methods 2023;3:100476. 10.1016/j.crmeth.2023.100476. 37323566 PMC10261906

[27] De Meo P, Ferrara E, Fiumara G. et al. Generalized Louvain method for community detection in large networks. 2011 11th International Conference on Intelligent Systems Design and Applications, 2011, 88–93. 10.1109/isda.2011.6121636.

[28] You Y, Chen T, Sui Y. et al. Graph contrastive learning with augmentations. Adv Neural Inf Process Syst 2020;33:5812–23.

[29] You Y, Chen T, Shen Y. et al. Graph contrastive learning automated. In: International Conference on Machine Learning, pp. 12121–32. The Thirty-Eighth International Conference on Machine Learning 2021, PMLR, 2021.

[30] Lan W, Yang T, Chen Q. et al. Multiview subspace clustering via low-rank symmetric affinity graph. IEEE Trans Neural Netw Learn Syst 2023;35:11382–95. 10.1109/TNNLS.2023.3260258. 37015132

[31] Lan W, Zhou G, Chen Q. et al. Contrastive clustering learning for multi-behavior recommendation. ACM Trans Inf Syst 2024;43:1–23.

[32] Chunyu , Wei Y, Wang BB, Ni K. et al. Boosting graph contrastive learning via graph contrastive saliency. In: International Conference on Machine Learning, PMLR, The Fortieth International Conference on Machine Learning 2023, pp. 36839–55. 2023.

[33] Sun M, Xing J. Wang H. et al. MoCL: data-driven molecular fingerprint via knowledge-aware contrastive learning from molecular graph. Proceedings of the 27th ACM SIGKDD Conference on Knowledge Discovery & Data Mining. 2021. 10.1145/3447548.3467186. PMC9105980 35571558

[34] Wang Y, Wang J, Cao Z. et al. Molecular contrastive learning of representations via graph neural networks. Nat Mach Intell 2022;4:279–87. 10.1038/s42256-022-00447-x.

[35] Wang Y, Min Y, Chen X. et al. Multi-view graph contrastive representation learning for drug-drug interaction prediction. Proc Web Conf 2021;2021:2921–33.

[36] Duan H, Zhang Q, Cui F. et al. MVST: Identifying spatial domains of spatial transcriptomes from multiple views using multi-view graph convolutional networks. PLoS Comput Biol 2024;20:e1012409. 10.1371/journal.pcbi.1012409. PMC11376559 39235988

[37] Jang E, Gu S, Poole B. Categorical reparameterization with Gumbel-Softmax. 5th International Conference on Learning Representations 2017. arXiv preprint arXiv:1611.01144. 2016.

[38] Murtagh F . Multilayer perceptrons for classification and regression. Neurocomputing 1991;2:183–97. 10.1016/0925-2312(91)90023-5.

[39] Chen T, Bian S, Sun Y. Graph Feature Networks. RLGM workshop at ICLR'19. arXiv preprint arXiv:1905.04579. 2019.

[40] Tian Y, Sun C, Poole B. et al. What makes for good views for contrastive learning? Adv Neural Inf Process Syst 2020;33:6827–39.

[41] Kusaba T, Lalli M, Kramann R. et al. Differentiated kidney epithelial cells repair injured proximal tubule. Proc Natl Acad Sci 2014;111:1527–32. 24127583 10.1073/pnas.1310653110 PMC3910580

[42] Smeets B, Boor P, Dijkman H. et al. Proximal tubular cells contain a phenotypically distinct, scattered cell population involved in tubular regeneration. J Pathol 2013;229:645–59. 10.1002/path.4125. 23124355 PMC3951144

[43] Fang Z, Liu X, Peltz G. GSEApy: a comprehensive package for performing gene set enrichment analysis in python. Bioinformatics 2023;39:btac757. 36426870 10.1093/bioinformatics/btac757 PMC9805564

[44] Kanehisa M, Furumichi M, Tanabe M. et al. KEGG: new perspectives on genomes, pathways, diseases and drugs. Nucleic Acids Res 2017;45:D353–61. 10.1093/nar/gkw1092. 27899662 PMC5210567

[45] Ietswaart R, Gyori BM, Bachman JA. et al. Genewalk identifies relevant gene functions for a biological context using network representation learning. Genome Biol 2021;22:1–35. 10.1186/s13059-021-02264-8. 33526072 PMC7852222

[46] Reimand J, Isserlin R, Voisin V. et al. Pathway enrichment analysis and visualization of omics data using g:Profiler, GSEA, Cytoscape and EnrichmentMap. Nat Protoc 2019;14:482–517. 10.1038/s41596-018-0103-9. 30664679 PMC6607905

[47] Moore JEC, Trager L, Anzia LE. et al. Dofetilide for suppression of atrial fibrillation in hypertrophic cardiomyopathy: a case series and literature review. Pacing Clin Electrophysiol 2018;41:396–401. 10.1111/pace.13310. 29450893

[48] Sakai T, Tsushima T, Kimura D. et al. Primary lung cancer associated with dilated phase of hypertrophic cardiomyopathy; report of a case. Kyobu geka 2017;70:147–50. 28174411

[49] Hoernes M, Seger R, Reichenbach J. Modern management of primary b-cell immunodeficiencies. Pediatr Allergy Immunol 2011;22:758–69. 10.1111/j.1399-3038.2011.01236.x. 22122788

[50] Wang H, Xizi Jiang Y, Cheng HR. et al. MZT2A promotes nsclc viability and invasion by increasing AKT phosphorylation via the MOZART2 domain. Cancer Sci 2021;112:2210–22. 10.1111/cas.14900. 33754417 PMC8177791

[51] Wang T, Huo X, Chong Z. et al. A review of S100 protein family in lung cancer. Clin Chim Acta 2018;476:54–9. 10.1016/j.cca.2017.11.010. 29146477

[52] Liu Y, Cui J, Tang Y-L. et al. Prognostic roles of mRNA expression of S100 in non-small-cell lung cancer. Biomed Res Int 2018;2018:9815806. 29607329 10.1155/2018/9815806 PMC5828052

[53] Ueda K, Arakawa H, Nakamura Y. Dual-specificity phosphatase 5 (DUSP5) as a direct transcriptional target of tumor suppressor P53. Oncogene 2003;22:5586–91. 10.1038/sj.onc.1206845. 12944906

[54] Buffet C, Hecale-Perlemoine K, Bricaire L. et al. DUSP5 and DUSP6, two ERK specific phosphatases, are markers of a higher MAPK signaling activation in BRAF mutated thyroid cancers. PLoS One 2017;12:e0184861. 10.1371/journal.pone.0184861. PMC5599027 28910386

[55] Joshi V, Lakhani SR, McCart Reed AE. NDRG1 in Cancer: A Suppressor, Promoter, or Both? Cancers 2022;14:5739. 10.3390/cancers14235739. PMC9737586 36497221

[56] Miskulin M, Dalgleish R, Kluve-Beckerman B. et al. Human type III collagen gene expression is coordinately modulated with the type I collagen genes during fibroblast growth. Biochemistry 1986;25:1408–13. 10.1021/bi00354a033. 3754462

