# Supplementary material for: Graph contrastive learning of subcellular-resolution spatial transcriptomics improves cell type annotation and reveals critical molecular pathways
Source: Brief Bioinform. 2025 Jan 30;26(1):bbaf020. doi: 10.1093/bib/bbaf020 (PMC11781232; doi:10.1093/bib/bbaf020)
Supplement: supplementary_bbaf020 [file supplementary_bbaf020.pdf]

Supplementary Materials

Additional ablation studies

Table 1. Ablation studies and hyperparameter analysis on MERFISH MOP datasets. (Bold text indicates the best, underlined text indicates the second-best, and 'N/A' means no meaningful results.)

Augmentation Level	Augmentation Type*	$\lambda = 0.5$		$\lambda = 1$		$\lambda = 5$	
		Accuracy	F1-score	Accuracy	F1-score	Accuracy	F1-score
**Node**	**Identical**	0.823±0.021	0.804±0.017	0.933±0.021	0.915±0.022	0.947±0.032	0.911±0.021
**Node**	**ND**	0.907±0.022	0.853±0.045	0.942±0.035	0.892±0.041	0.970±0.015	0.961±0.018
**Node**	**NM**	0.932±0.014	0.885±0.028	0.944±0.022	0.898±0.033	0.954±0.009	0.922±0.010
**Node**	**EP**	0.872±0.019	0.869±0.035	0.901±0.036	0.871±0.025	0.967±0.021	0.944±0.011
**Node**	**ND & NM**	**0.955±0.014**	0.901±0.012	0.960±0.009	0.911±0.004	0.944±0.014	0.887±0.025
**Node**	**ND, NM & EP**	0.882±0.020	0.828±0.021	0.934±0.024	0.912±0.009	0.965±0.017	0.921±0.023
**Subgraph**	**EP**	0.897±0.016	0.811±0.018	0.927±0.046	0.887±0.082	0.937±0.033	0.889±0.029
**Subgraph**	**SW**	0.941 ±0.023	0.861 ±0.021	0.933±0.008	0.902±0.006	0.945±0.018	0.889±0.019
**Subgraph**	**EP & SW**	0.942±0.029	0.899±0.021	0.911±0.014	0.882±0.014	0.941±0.026	0.885±0.024
**Node & Subgraph**	**ALL**	**0.955±0.012**	**0.902±0.026**	**0.964±0.013**	**0.913±0.030**	**0.975±0.011**	**0.967±0.018**

* Identical: no augmentation; ND: node dropping; NM: node masking; EP: edge perturbation; SW: subgraph swapping; ALL: all augmentations including ND, NM & EP from node level and EP & SW from subgraph level.

Table 2. Performance comparison on CosMx Lung, Xenium DCIS, MERFISH MOp datasets using different GNN modules. CosMx Lung: reference dataset ID: 5-2; query dataset ID: 5-3; Xenium DCIS: reference dataset ID: replicate2 ; query dataset ID: replicate1; MERFISH MOp: reference dataset ID: m12; query dataset ID: m22 (Bold indicates the best, underlined the second-best.)

Model Type	CosMx Lung		Xenium DCIS		MERFISH	
	Accuracy	F1-score	Accuracy	F1-score	Accuracy	F1-score
**ResGCN**	**0.901±0.021**	**0.712±0.008**	**0.867±0.019**	**0.734±0.021**	**0.975±0.011**	**0.967±0.018**
**GIN**	0.817±0.041	0.634±0.044	0.802±0.039	0.688±0.043	0.857±0.035	0.825±0.028
**GCN**	0.856±0.021	0.695±0.017	0.843±0.018	0.729±0.011	0.924±0.015	0.901±0.018
**GAT**	0.847±0.010	0.702±0.007	0.856±0.006	0.704±0.004	0.926±0.007	0.899±0.011
**GraphSAGE**	0.867±0.012	0.711±0.009	0.863±0.008	0.733±0.004	0.929±0.007	0.869±0.003

Additional experiments using different subgraph clustering methods

Table 3. Performance comparison of subgraph clustering methods.

Experiment Setting	Datasets (Reference/Query)	Focus(Louvain)		Focus(Leiden)		Focus(LPA)	
		Accuracy	F1-score	Accuracy	F1-score	Accuracy	F1-score
CosMx Lung(Same)	5-2 / 5-1	0.899	**0.671**	**0.898**	0.666	0.853	0.664
	5-3 / 5-1	**0.928**	**0.732**	0.900	0.709	0.888	0.687
	5-1 / 5-2	0.886	**0.689**	**0.892**	0.652	0.869	0.631
CosMx Lung(Diff)	12 / 5-1	0.751	**0.493**	0.747	0.492	**0.748**	0.484
	12 / 5-2	**0.791**	0.520	0.778	0.501	**0.791**	**0.521**
	12 / 5-3	0.786	0.508	**0.797**	**0.512**	0.761	0.496
CosMx Kidney(Same)	4061 / 10838	0.616	0.608	0.683	0.683	**0.690**	**0.695**
	1098 / 10838	**0.702**	**0.701**	0.661	0.683	0.686	0.682
	10838 / 1098	0.658	0.654	0.664	0.664	**0.671**	**0.687**
CosMx Kidney(Diff)	2566 / 10838	**0.697**	**0.687**	0.646	0.647	0.674	0.649
	3323 / 10838	**0.732**	**0.722**	0.672	0.676	0.680	0.694
MERFISH MOp(Same)	m11 / m12	**0.932**	**0.851**	0.910	0.843	0.899	0.848
	m21 / m22	**0.965**	**0.967**	0.944	0.921	0.944	0.912
MERFISH MOp(Diff)	m11 / m22	**0.927**	0.831	0.921	**0.843**	0.884	0.822
	m12 / m22	0.940	0.887	**0.942**	0.887	0.941	**0.900**
Xenium DCIS(Same)	replicate1 / replicate2	**0.876**	0.691	0.862	0.744	0.853	**0.745**
	replicate2 / replicate1	0.867	**0.734**	**0.865**	0.726	0.862	**0.734**

Note : Same: Reference and query datasets are from the same patient or mouse; Diff: Reference and query datasets are from different patients or mice

Detailed performance comparison of models on diverse datasets.

Table 4. Performance comparison of models on CosMx Lung datasets. The reference and query samples come from the same patient. (Bold indicates the best, underlined the second-best.)

Dataset ID	5-2 / 5-1		5-3 / 5-1		5-1 / 5-2		5-3 / 5-2		5-1 / 5-3		5-2 / 5-3	
Model	Accuracy	F1-score	Accuracy	F1-score	Accuracy	F1-score	Accuracy	F1-score	Accuracy	F1-score	Accuracy	F1-score
Focus	**0.899**	<u>0.671</u>	**0.928**	**0.732**	<u>0.886</u>	<u>0.689</u>	<u>0.903</u>	<u>0.717</u>	<u>0.899</u>	<u>0.708</u>	<u>0.902</u>	**0.712**
scDeepSort	0.614	0.318	0.756	0.437	0.766	0.426	0.748	0.421	0.813	0.443	0.651	0.301
CellTypist	0.631	0.429	0.639	0.433	0.599	0.452	0.589	0.388	0.692	0.460	0.657	0.410
TOSICA	0.832	0.622	0.837	0.632	0.872	0.650	0.855	0.648	0.864	0.655	0.861	0.623
ACTTINN	0.857	0.542	0.835	0.567	0.857	0.602	0.842	0.556	0.863	0.540	0.855	0.584
Tacco	**0.899**	**0.699**	<u>0.909</u>	<u>0.718</u>	**0.925**	**0.765**	**0.918**	**0.745**	**0.930**	**0.742**	**0.916**	<u>0.704</u>
scDot	0.834	0.612	0.827	0.653	0.844	0.627	0.867	0.666	0.844	0.610	0.847	0.653

Table 5. Performance comparison of models on CosMx Lung datasets. The reference and query samples come from different patients. (Bold indicates the best, underlined the second-best.)

Dataset ID	12 / 5-2		12 / 5-3		13 / 5-2		13 / 5-3	
Model	Accuracy	F1-score	Accuracy	F1-score	Accuracy	F1-score	Accuracy	F1-score
Focus	**0.791**	**0.520**	**0.797**	**0.512**	**0.560**	**0.288**	**0.622**	**0.275**
scDeepSort	0.424	0.120	0.483	0.117	0.433	0.177	0.508	0.184
CellTypist	0.362	0.190	0.455	0.222	0.461	0.227	0.521	0.216
TOSICA	0.787	0.498	<u>0.781</u>	0.498	0.467	0.272	0.502	**0.275**
ACTTINN	0.681	0.346	0.714	0.342	<u>0.533</u>	0.242	<u>0.614</u>	0.266
Tacco	0.703	0.464	0.748	0.486	0.517	0.241	0.589	0.258
scDot	<u>0.790</u>	<u>0.517</u>	0.761	<u>0.503</u>	0.500	<u>0.270</u>	0.601	0.271

Table 6. Performance comparison of models on CosMx Kidney datasets. The reference/query dataset ID(4061/10838, 1098/10838, 10838/1098) comes from the same patient while the reference/query dataset ID(2566/10838, 3323/10838) comes from the different patients. (Bold indicates the best, underlined the second-best.)

Dataset ID	4061 / 10838		1098 / 10838		10838 / 1098		2566 / 10838		3323 / 10838	
Model	Accuracy	F1-score	Accuracy	F1-score	Accuracy	F1-score	Accuracy	F1-score	Accuracy	F1-score
Focus	**0.690**	**0.695**	<u>0.702</u>	<u>0.701</u>	**0.671**	**0.687**	<u>0.697</u>	<u>0.694</u>	**0.732**	**0.722**
scDeepSort	0.278	0.169	0.582	0.395	0.271	0.179	0.257	0.109	0.089	0.021
CellTypist	0.438	0.476	0.518	0.548	0.478	0.462	0.496	0.515	0.403	0.428
TOSICA	<u>0.641</u>	<u>0.644</u>	**0.724**	**0.707**	<u>0.638</u>	<u>0.641</u>	**0.733**	**0.715**	<u>0.707</u>	<u>0.691</u>
ACTTINN	0.563	0.543	0.592	0.560	0.551	0.551	0.591	0.559	0.578	0.527
Tacco	0.518	0.499	0.596	0.591	0.476	0.476	0.609	0.601	0.540	0.522
scDot	0.433	0.461	0.616	0.643	0.394	0.414	0.627	0.603	0.540	0.505

Table 7. Performance Comparison of Models on MERFISH MOp datasets. The reference/query dataset ID(m11/m12, m21/m22) comes from the same mouse while the reference/query dataset ID(m11/m22, m12/m22) comes from the different mice. (Bold text indicates the best, underlined text indicates the second-best, and 'N/A' means no meaningful results.)

**Dataset ID**	**m11 / m12**		**m21 / m22**		**m11 / m22**		**m12 / m22**	
**Model**	**Accuracy**	**F1**	**Accuracy**	**F1**	**Accuracy**	**F1**	**Accuracy**	**F1**
**Focus**	0.932	0.851	**0.965**	**0.951**	**0.927**	**0.831**	**0.941**	**0.900**
**scDeepSort**	0.831	0.667	0.904	0.702	0.83	0.63	0.877	0.73
**CellTypist**	N/A	N/A	N/A	N/A	N/A	N/A	N/A	N/A
**TOSICA**	**0.939**	<u>0.860</u>	0.945	0.920	0.921	0.821	0.929	0.829
**ACTTINN**	0.930	0.811	0.946	0.895	<u>0.925</u>	0.804	<u>0.936</u>	0.859
**Tacco**	<u>0.936</u>	**0.868**	<u>0.948</u>	<u>0.935</u>	0.915	<u>0.826</u>	0.930	0.874
**scDot**	0.901	0.811	0.942	0.924	0.917	0.813	0.918	<u>0.880</u>

Table 8. Performance comparison of models on Xenium DICS datasets. The reference/query dataset ID(replicate1/replicate2, replicate2/replicate1) comes from the same patient. (Bold text indicates the best, underlined text indicates the second-best, and 'N/A' means no meaningful results.)

**Dataset ID**	**replicate1 / replicate2**		**replicate2 / replicate1**	
**Model**	**Accuracy**	**F1-score**	**Accuracy**	**F1-score**
**Focus**	**0.876**	**0.691**	**0.867**	**0.734**
**scDeepSort**	N/A	N/A	N/A	N/A
**CellTypist**	0.363	0.142	0.379	0.145
**TOSICA**	0.350	0.179	0.412	0.172
**ACTTINN**	<u>0.602</u>	0.196	<u>0.584</u>	0.190
**Tacco**	0.524	<u>0.212</u>	0.525	<u>0.202</u>
**scDot**	N/A	N/A	N/A	N/A

Loss Plot

Figure 1 provides a comprehensive overview of the training process of the Focus model on MERFISH MOp datasets (Reference dataset: m21; Query dataset: m22). Subfigure (a) illustrates the total loss decreasing steadily over epochs, reflecting the convergence of the training process. Subfigure (b) highlight the classification losses for the query dataset, respectively, showing consistent improvement in cell type classification accuracy over epochs. Subfigure (c) tracks the accuracy of the query dataset, reflecting consistent gains in balanced classification performance.(d) depicts the F1-score of the query dataset, which follows a similar upward trend, indicating effective model adaptation to the query dataset. Together, these plots provide a detailed representation of the model's performance and optimization during training.

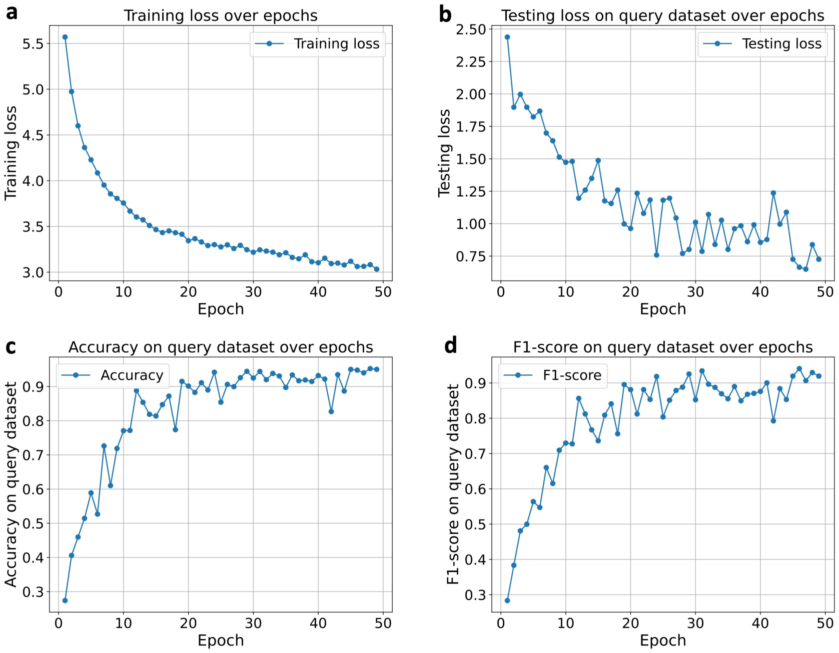

Figure 1. Training process of Focus on MERFISH MOp datasets (Reference dataset ID: m21; Query dataset ID: m22) a. Training loss (contrastive loss + classification loss) over epochs. **b**. Testing loss (classification loss) on query dataset over epochs. **c**. Accuracy on query dataset over epochs. **d**. F1-score on query dataset over epochs.
